# Codevelopment of gut microbial metabolism and visual neural circuitry over human infancy

**DOI:** 10.1128/mbio.00835-25

**Published:** 2025-06-30

**Authors:** Kevin S. Bonham, Emma T. Margolis, Guilherme Fahur Bottino, Ana C. Sobrino, Fadheela Patel, Shelley McCann, Michal R. Zieff, Marlie Miles, Donna Herr, Lauren Davel, Cara Bosco, Khula South African Data Collection Team, Layla Bradford, Curtis Huttenhower, Nicolò Pini, Daniel C. Alexander, Derek K. Jones, Steve C. R. Williams, Dima Amso, Melissa Gladstone, William P. Fifer, Kirsten A. Donald, Laurel J. Gabard-Durnam, Vanja Klepac-Ceraj

**Affiliations:** 1 Department of Biological Sciences, Wellesley College 189352, Wellesley, Massachusetts, USA; 2 Department of Medicine, Tufts Medical Center 1867, Boston, Massachusetts, USA; 3 Department of Psychology, Northeastern University 1848, Boston, Massachusetts, USA; 4 Division of Medical Microbiology, University of Cape Town 37716, Cape Town, Western Cape, South Africa; 5 Department of Paediatrics and Child Health, University of Cape Town 37716, Cape Town, Western Cape, South Africa; 6 Department of Biostatistics, Harvard T. H. Chan School of Public Health 1857 , Boston, Massachusetts, USA; 7 Department of Psychiatry, Columbia University, Irving Medical Center 5798, , New York, New York, USA; 8 Division of Developmental Neuroscience, New York State Psychiatric Institute 27424, New York, New York, USA; 9 Department of Computer Science, Centre for Medical Image Computing, University College London 4919, London, England, United Kingdom; 10 Cardiff University Brain Research Imaging Centre, Cardiff University 2112, Cardiff, United Kingdom; 11 Department of Neuroimaging, King's College London 4616, London, England, United Kingdom; 12 Department of Psychology, Columbia University 5798, New York, New York, USA; 13 Department of Women and Children’s Health, Institute of Life Course and Medical Science, Alder Hey Children’s NHS Foundation Trust University of Liverpool 4591, Liverpool, England, United Kingdom; 14 Neuroscience Institute, University of Cape Town 698896, Cape Town, Western Cape, South Africa; University of Maryland School of Medicine, Baltimore, Maryland, USA

**Keywords:** visual cortex development, metagenome, infant gut microbiome, visual-evoked potential, neuroplasticity

## Abstract

Infancy is a time of elevated neuroplasticity supporting rapid brain and sensory development. The gut microbiome, also undergoing extensive developmental changes in early life, may influence brain development through the metabolism of neuroactive compounds. Here, we leverage longitudinal data from 194 South African infants across the first 18 months of life to show that microbial genes encoding enzymes that metabolize molecules playing a key role in modulating early neuroplasticity are associated with visual cortical neurodevelopment, measured by the Visual-Evoked Potential (VEP). Neuroactive compounds included neurotransmitters GABA and glutamate, the amino acid tryptophan, and short-chain fatty acids involved in myelination, including acetate and butyrate. Microbial gene sets around 4 months of age were strongly associated with the VEP from around 9–14 months of age and showed more associations than concurrently measured gene sets, suggesting that microbial metabolism in early life may affect subsequent neural plasticity and development.

**IMPORTANCE:**

Over the past decade, extensive research has revealed strong links between the gut microbiome and the brain, at least in adults or those with neuropsychiatric disorders. This study explores how these associations emerge in early development using a longitudinal sample of 194 infants with repeated microbiome metabolism and electroencephalography (EEG) measures during the critical early period of visual cortex neuroplasticity. We examined microbial genes encoding enzymes for neuroactive compounds (e.g., GABA, glutamate, tryptophan, and short-chain fatty acids) and their association with the visual-evoked potential (VEP). Genes from 4-month stool samples strongly correlated with VEP features between 9 and 14 months, suggesting that early microbial metabolism influences later visual neurodevelopment. These prospective associations were more numerous than the concurrent ones. Our findings suggest that early gut microbiome metabolic potential plays a crucial role in shaping neural plasticity and visual neurodevelopment.

## INTRODUCTION

The gut microbiome in early life has potential long-term implications for brain and body health. One important way this influence can occur is through interactions with the central nervous system as a “microbial-gut-brain axis” (1–3). The metabolic potential of the microorganisms that inhabit the gut vastly exceeds that of human cells alone, with microbial genes outnumbering host genes by a hundredfold (4). In particular, gut microbes have the ability to metabolize and synthesize many neuroactive compounds (5). However, the physiological relevance of this in humans has been difficult to quantify, particularly during initial neurological development in early life.

Extensive work in preclinical models suggests that these neuroactive compounds can influence the brain through both direct and indirect pathways. For example, major neurotransmitters (e.g., glutamate, γ-aminobutyric acid [GABA], serotonin, and dopamine) are readily synthesized and degraded by intestinal microbes and can enter circulation and pass the blood-brain barrier to influence central nervous function (6–9). Glutamatergic/GABA-ergic signaling is critical for balancing the brain’s excitatory and inhibitory neurotransmission levels, and alterations in the bi-directional glutamatergic/GABA-ergic signaling between the gut microbiome and brain are implicated in several physical and mental health conditions (10, 11). Similarly, the gut and the microbiome are critical to the regulation of metabolism for the neurotransmitters serotonin and dopamine, particularly through the metabolism of dietary tryptophan (12). Moreover, short-chain fatty acids (SCFAs) produced by the gut microbiome may impact the brain directly by modulating neurotrophic factors, glial and microglial maturation and myelination, and neuroinflammation (13, 14). Other indirect pathways for gut microbial influence on the brain include vagus nerve stimulation, neuroendocrine modulation, and immune system regulation (1).

Rapidly growing literature connects the metabolic potential of the gut microbiome and brain function in humans (reviewed in [7, 15, 16]), but the overwhelming majority of this research is conducted in adult participants. Importantly, both the gut microbiome and the brain undergo dramatic and rapid development over the first postnatal years (17–19). However, very little is currently known about how gut-brain influences emerge or change during this critical window (20–22). Interrogating this early co-development in humans is key to both understanding adaptive gut-brain function and behavior and informing strategies to support it. Specifically, the visual cortex has been shown to be sensitive to gut microbiome modulations in adults (23) and rodents (24); however, the visual cortex undergoes its most rapid period of plasticity and maturation over infancy at the same time the microbiome changes most significantly (25–27).

Visual cortical maturation can be robustly indexed via electroencephalography (EEG) with the visual-evoked potential (VEP) response to visual stimuli from birth. The VEP is an important paradigm for indexing neurodevelopment, given its translational potential, as it can be studied mechanistically across species and has clinical utility (28, 29). It is an especially useful index of ongoing maturation, as it includes amplitude deflections—reflecting underlying cortical circuit function (i.e., the balance of excitatory/inhibitory postsynaptic potentials [18, 30, 31])—as well as latencies to those deflections, which are thought to reflect structural integrity and myelination of the parvocellular and magnocellular pathways (18, 29, 32, 33). The VEP includes three components to be quantified: the N1 (first negative: going deflection), P1 (first positive: going deflection), and the N2 (second negative: going deflection). The N1 and N2 components, which come online in 3 months (18), are generated by the parvocellular visual pathway, which is most sensitive to color and spatial detail, whereas the P1 component, present from birth, is generated by the magnocellular pathway, which is most sensitive to motion (34–39). The overall VEP morphology stabilizes, reflecting adult-like patterns, in the second year of life (18).

Here, we investigated the longitudinal co-development of microbial metabolic potential quantified via genes encoding enzymes that metabolize neuroactive compounds and visual neurodevelopment as indexed by the VEP in a longitudinal community sample of 194 infants from Gugulethu in Cape Town, South Africa, recruited as part of the prospective longitudinal “Khula” Study (40). Stool samples and EEG were each collected at up to three visits in the first 18 months of life. Shotgun metagenomic sequencing was used to obtain microbial gene sequences from infant stool samples. To index visual cortical functional development, latencies and peak amplitudes were extracted from each component of the VEP, producing six VEP features of interest. We evaluated the concurrent association between microbial genes and VEP amplitudes and latencies, and we tested prospective influences of microbial genes from early visits on VEP changes at later visits. In this way, we were able to reveal the temporal dynamics of gut-brain co-development within individuals during this most critical window of plasticity in both systems.

## MATERIALS AND METHODS

### Cohort

#### Participants and study design

**Fig 1:**
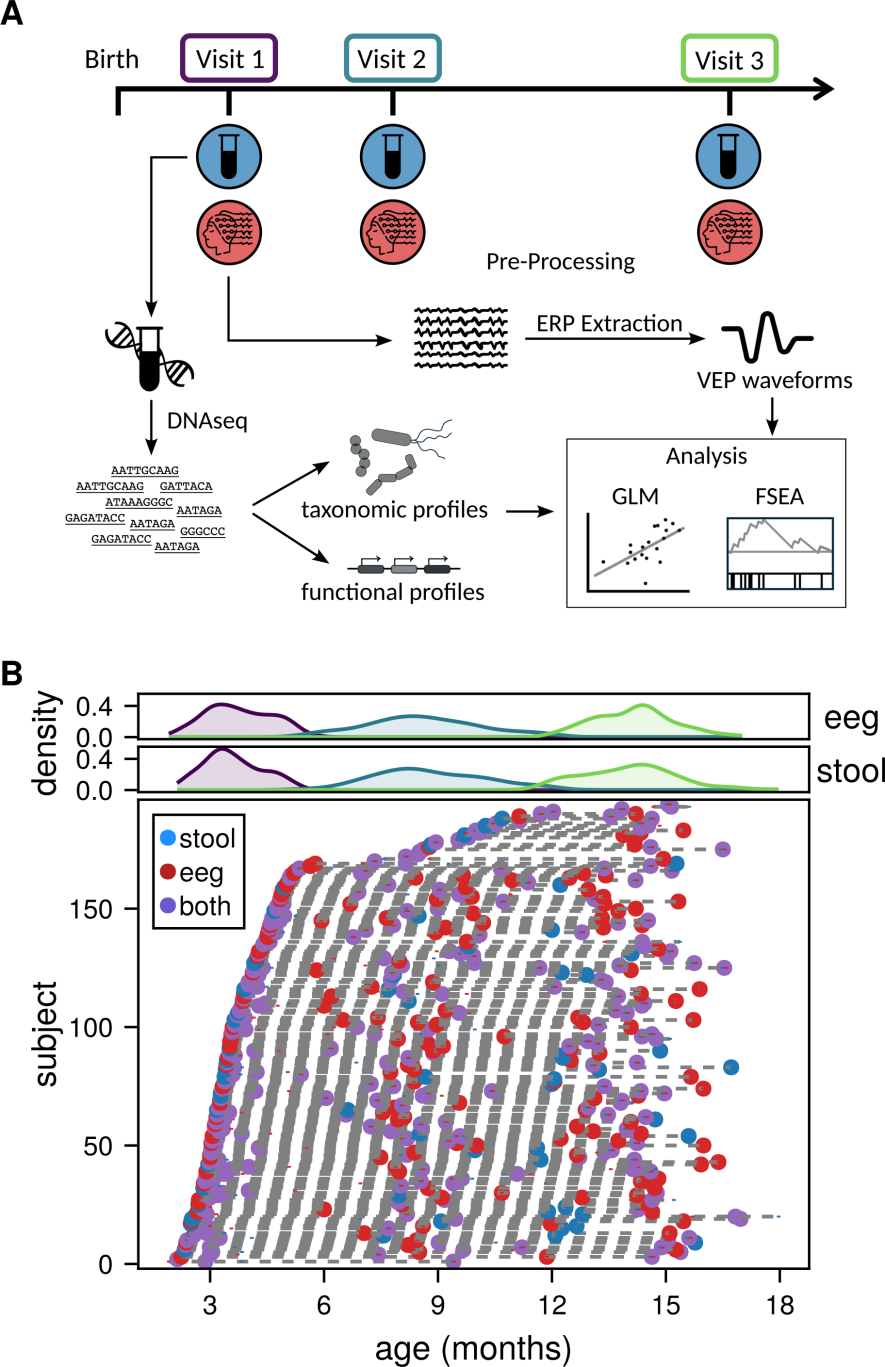
Study design to capture the dynamic nature of early microbiome and brain development. (**A**) Study design; participants (*N* = 194) were seen up to three times over the first 18 months of life. Stool samples and EEG data were collected, generating microbial functional profiles (stool) and VEP waveforms (EEG) used in subsequent analyses. Analyses included generalized linear models (GLM) and feature set enrichment analysis (FSEA). (**B**) Longitudinal sampling of study participants; density plots (top) for stool and EEG collection show the ages represented in each visit. The scatter plot (bottom) shows individual participant visits. Dotted lines connect separate visits for the same participant. When stool and EEG data were collected for the same visit (purple) but not on the same day, the dot represents the median age of collection, and vertical bars in blue and red represent stool and EEG collections, respectively.

Infants were recruited from the local community clinics in Gugulethu, an informal settlement in Cape Town, South Africa, as part of a prospective longitudinal study (most enrollments happened prenatally with 16% of infants enrolled shortly after birth; 40). The first language of the majority of residents in this area is Xhosa. Study procedures were offered in English or Xhosa, depending on the language preference of the mother. This study was approved by the relevant university Health Research Ethics Committees (University of Cape Town study number: 666/2021). Informed consent was collected from mothers on behalf of themselves and their infants. Demographic information, including maternal place of birth, primary spoken language, maternal age at enrollment, maternal educational attainment, and maternal income, was collected at enrollment (Fig. 1, Table 1).

**TABLE 1:** Overall demographic information 14.1 (1.03)

	Overall (*N* = 194)
Mean (SD) age at EEG data collection (months)	
Visit 1 (*N* = 97)	3.7 (0.85)
Visit 2 (*N* = 129)	8.6 (1.46)
Visit 3 (*N* = 130)	14.1 (1.03)
Mean (SD) age at stool data collection (months)	
Visit 1 (*N* = 119)	3.6 (0.76)
Visit 2 (*N* = 105)	8.8 (1.43)
Visit 3 (*N* = 91)	14.0 (1.24)
Maternal place of birth	
South Africa	191 (98.5%)
In the African continent (not South Africa)	3 (1.5%)
Primary spoken language	
Xhosa Language	187 (96.4%)
Sotho Language	2 (1.0%)
Zulu Language	1 (0.5%)
English Language	2 (1.0%)
Ndebele Language	1 (0.5%)
Afrikaans Language	1 (0.5%)
Maternal Age at Infant Birth (years)	
Mean (SD)	29.2 (5.63)
Median [Min, Max]	29.0 [18.0, 41.0]
Missing	1 (0.5%)
Maternal educational attainment^*a*^	
Completed Grade 6 (Standard 4) to Grade 7 (Standard 5)	4 (2.1%)
Completed Grade 8 (Standard 6) to Grade 11 (Standard 9), i.e., high school without matriculating	78 (40.2%)
Completed Grade 12 (Standard 10) i.e., high school	88 (45.4%)
Part of university/college/post-matric education	13 (6.7%)
Completed university/ college/ post-matric education	11 (5.7%)
Maternal monthly income^*b*^ (South African Rand/ZAR)	
Less than R1000 per month	97 (50.0%)
R1000 to R5000 per month	76 (39.2%)
R5000 to R10,000 per month	16 (8.2%)
More than R10,000 per month	0 (0%)
Unknown	5 (2.6%)
Infant Biological Sex	
Female	91 (46.9%)
Male	103 (53.1%)

^*a*^ The South African Educational System was formerly divided into years called standards, similar to the way the United States Educational System is divided into grades. The equivalent in terms of standards is provided in parentheses next to each mentioned grade. “University/College/Post-Matric Education” refers to tertiary or post-secondary education as defined by the World Bank.

^*b*^ At the time of writing (1/16/24), 1 US Dollar = 18.87 South African Rand (ZAR).

Families were invited to participate in three in-lab study visits over their infant’s first 2 years of life. At the first in-lab study visit (hereafter visit 1), occurring when infants were between approximately 2 months and 6 months of age, (age in months: M = 3.7, SD = 0.85, range = 1.91–5.54), the following data were collected: the infant age (in months), sex, infant electroencephalography (EEG), and infant stool samples.

At the second study visit (hereafter visit 2), occurring when infants were between approximately 6 months and 12 months of age (age in months: M = 8.60, SD = 1.48, range = 5.41–12.00), and the third study visit (hereafter visit 3), occurring when infants were between approximately 12 months and 17 months of age (age in months: M = 14.10, SD = 1.04, range = 12.10–17.00), infant EEG and stool samples were collected again. These visits were selected to capture the visual-evoked potential’s dynamic course of maturation in early life: the transition from a predominantly single positive wave (i.e., P1) to a clearly triphasic waveform with the N1 and N2 components around 3 months, marked latency reductions in the first 7 months, and continued maturation with an adult-like pattern emerging in the second year of life (18). At visits in which infants were unable to complete both EEG and stool samples on the same day, EEG and stool samples were collected on different days. For concurrent time point analyses, infants with EEG and stool collected more than 2 months apart were excluded. Not all infants had EEG and microbiome data collected at all three time points or contributed usable data at all three time points.

All enrolled infants received a comprehensive medical exam at each visit, which included assessments of eye-related conditions. Several infants (*n* = 3) were identified as having eye-related anomalies during the medical exam, and they were excluded from any further analyses.

### EEG processing

#### EEG data acquisition

Electroencephalography (EEG) data were acquired from infants while they were seated in their caregiver’s lap in a dimly-lit, quiet room using a 128-channel high-density HydroCel Geodesic Sensor Net (EGI, Eugene, OR), amplified with a NetAmps 400 high-input amplifier, and recorded via an Electrical Geodesics, Inc. (EGI, Eugene, OR) system with a 1,000 Hz sampling rate. EEG data were online referenced to the vertex (channel Cz) using EGI Netstation software. Impedances were kept below 100 KΩ in accordance with the impedance capabilities of the high-impedance amplifiers. Geodesic Sensor Nets with modified tall pedestals designed to improve the inclusion of infants with thick/curly/tall hair were used as needed across participants (41). Shea Moisture leave-in castor oil conditioner was applied to hair across the scalp prior to net placement to improve both impedances and participant comfort (41). This leave-in conditioner contains insulating ingredients; hence, there is no risk of electrical bridging, and it has not been found to disrupt the EEG signal during testing (unpublished data). Conditioning hair in this way allows for nets to lay closer to the scalp for curly/coily hair types and makes for more comfortable net removal at the end of testing.

The visual-evoked potential (VEP) task was presented using Eprime 3.0 software (Psychology Software Tools, Pittsburgh, PA) on a Lenovo desktop computer with an external monitor 19.5 inches on the diagonal facing the infant (with a monitor approximately 65 cm away from the infant). A standard phase-reversal VEP was induced with a black and white checkerboard (1 × 1 cm squares within the board) stimulus that alternated presentation (black squares became white, white squares became black) every 500 ms for a total of 100 trials. Participants looking were monitored by video and by an assistant throughout data collection. If the participant looked away during the VEP task, the task was rerun.

#### EEG data pre-processing

VEP data were exported from native Netstation .mff format to .raw format and then pre-processed using the HAPPE + ER pipeline within HAPPE v3.3 software, an automated open-source EEG processing software validated for infant data (42). A subset of the 128 channels was selected for pre-processing that excluded the rim electrodes, as these are typically artifact-laden (channels excluded from pre-processing included in Table S1). The HAPPE pre-processing pipeline was run with user-selected specifications outlined in Table S1.

Pre-processed VEP data were considered usable and moved forward to VEP extraction if HAPPE pre-processing ran successfully, at least 15 trials were retained following bad trial rejection, and at least one good channel was kept within the visual ROI. Note that channels marked badly during pre-processing had their data interpolated as part of standard preprocessing pipelines for ERPs (42).

Interpolated channels were included in analyses here as is typically done in developmental samples, and given the low overall rates of interpolation present (e.g., an average of between 4 and 5 of 5 possible good channels in the region of interest were retained at each visit time point).

#### Visual-evoked potentials (VEPs)

VEP waveforms were extracted and quantified using the HAPPE + ER v3.3 GenerateERPs script (42). Electrodes in the occipital region were selected as a region of interest (i.e., E70, E71, E75, E76, and E83). The VEP waveform has three main components to be quantified: a negative N1 peak, a positive P1 peak, and a negative N2 peak. The windows for selecting the calculated features were based on preliminary visualizations of the waveforms at each visit, such that the selected windows would capture the most component peaks across all subjects. Due to normative maturation of the waveforms as infants age, one set of user-specified windows for calculating component features was used for visits 1 and 2, and another was used for visit 3. For visits 1 and 2, the window for calculating features for the N1 component was 40–100 ms, 75–175 ms for the P1 component, and 100–325 ms for the N2 component. For visit 3, the window for calculating features for the N1 component was 35–80 ms, 75–130 ms for the P1 component, and 100–275 ms for the N2 component. All VEPs were visually inspected to ensure that the automatically extracted values were correct and were adjusted if observable peaks occurred outside the automated window bounds. These visual checks ensure that peak amplitudes and latencies capture individual variability within and across visits. Participants were considered to have failed this visual inspection and were subsequently removed from the data set if their VEP did not produce three discernible peaks. HAPPE + ER parameters used in extracting the ERPs are summarized in Table S2. To correct for the potential influence of earlier components on later components, corrected amplitudes and latencies were calculated and used in all analyses. Specifically, the P1 amplitude was corrected for the N1 amplitude (corrected P1 amplitude = P1 N1 amplitude), the P1 latency was corrected for the N1 latency (corrected P1 latency = P1 N1 latency), the N2 amplitude was corrected for the P1 amplitude (corrected N2 amplitude = N2 P1 amplitude), and the N2 latency was corrected for the P1 latency (corrected N2 latency = N2 P1 latency).

**Fig 2:**
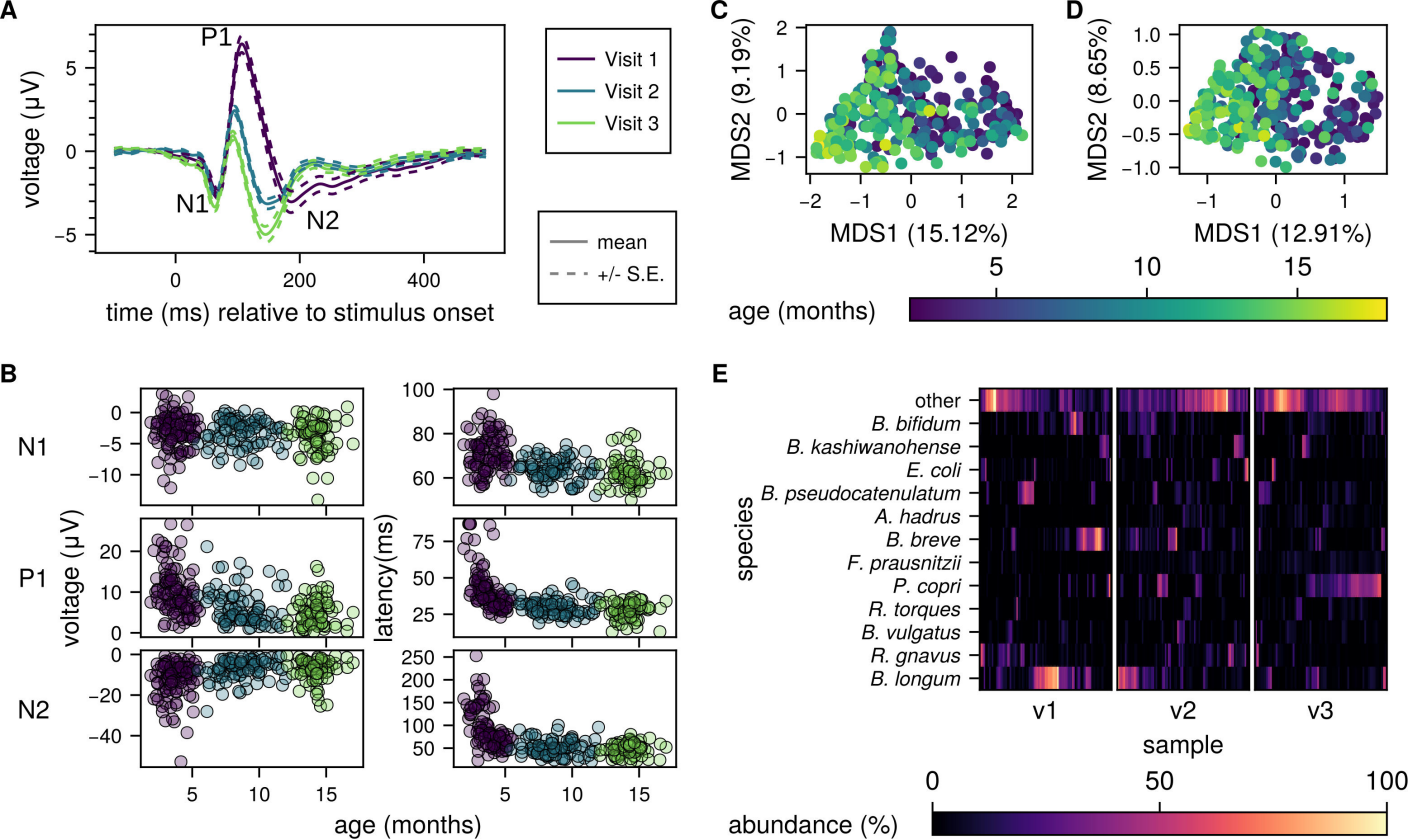
The gut microbiome and VEP both develop over the first 18 months of life. (**A**) Mean ± standard error of VEP curves from all included individuals at each visit. (**B**) Individual VEP feature measurements for peak amplitudes (left) and latencies (right) for all participants and all visits in the study, separated by age and colored by the visit as in (**A**). (**C**) Principal coordinate analysis (PCoA) by multidimensional scaling (MDS) on Bray-Curtis dissimilarity of taxonomic profiles; percent variance explained (fraction of positive eigenvalues) by each of the first two axes is indicated on the x and y axes, respectively. (**D**) PCoA of microbial functional profiles (UniRef90s). (**E**) Relative abundance of dominant bacterial taxa across three study visits. Heatmap showing the relative abundance (%) of the top 12 bacterial taxa (rows) detected in participant stool samples across three longitudinal visits (v1, v2, and v3; columns). Each column within a visit represents an individual sample from a participant, and samples are ordered by hierarchical clustering. “Other” represents the sum of all taxa not in the top 12. The X-axis within each visit is sorted by hierarchical clustering, and the y-axis is sorted by total abundance across all samples.

VEP waveforms of the included participants by time point are included in Fig. 2A. Ninety-seven infants provided usable VEP data at visit 1, 130 infants provided usable VEP data at visit 2, and 131 infants provided usable VEP data at visit 3. For included participants, EEG data quality metrics are summarized in Table S3. *t*-tests for data quality metrics (i.e., number of trials collected, number of trials retained, number of channels retained in the ROI, and Pearson’s r for data pre- vs. post-wavelet thresholding at 5, 8, 12, and 20 Hz) were run between each visit combination (i.e., visit 1 vs. visit 2, visit 1 vs. visit 3, and visit 2 vs. visit 3). For visits that differed in data quality, follow-up post hoc correlations were run for the data quality measure with each VEP feature at each visit in the *t*-test. In no case did the data quality metric relate to VEP features at multiple visits, making it highly unlikely the data quality difference contributed to results.

### Biospecimens and sequencing

#### Sample collection

Stool samples (*n* = 315) were collected in the clinic by the research assistant directly from the diaper, transferred to Zymo DNA/RNA ShieldTM Fecal collection Tubes (#R1101, Zymo Research Corp., Irvine, USA), and immediately frozen at −80 ˚C. Stool samples were not collected if the participant had taken antibiotics within the 2 weeks prior to sampling.

#### DNA extraction

DNA extraction was performed at Medical Microbiology, University of Cape Town, South Africa, from stool samples collected in DNA/RNA Shield Fecal collection tube using the Zymo Research Fecal DNA MiniPrep kit (# D4300, Zymo Research Corp., Irvine, USA) following the manufacturer’s protocol. To assess the extraction process’s quality, ZymoBIOMICS Microbial Community Standards (#D6300 and #D6310, Zymo Research Corp., Irvine, USA) were incorporated and subjected to the same process as the stool samples. The DNA yield and purity were determined using the NanoDrop ND−1000 (Nanodrop Technologies Inc. Wilmington, USA).

#### Sequencing

Shotgun metagenomic sequencing was performed on all samples at the Integrated Microbiome Research Resource (IMR, Dalhousie University, NS, Canada). A pooled library (max 96 samples per run) was prepared using the Illumina Nextera Flex Kit for MiSeq and NextSeq from 1 ng of each sample. Samples were then pooled onto a plate and sequenced on the Illumina NextSeq 2000 platform using 150 + 150 bp paired-end P3 cells, generating 24 M million raw reads and 3.6 Gb of sequence per sample (43).

#### Final sample sizes across analyses

After data processing and cleaning, analyses of concurrent EEG and microbiome data included 97 infants for visit 1, 86 infants for visit 2, and 70 infants for visit 3. For predictive analyses, 84 infants were included in the analyses of visit 1 stool on visit 2 EEG, 76 infants were included for visit 1 stool on visit 3 EEG, and 69 infants were included for visit 2 stool on visit 3 EEG.

### Statistics/computational analysis

#### Age-related changes in VEP features

To determine age-related changes in VEP features, six linear mixed models with each VEP feature as the outcome (i.e., N1 amplitude/latency, P1 amplitude/latency, N2 amplitude/latency) were run using the lme4 package (44) in R with age in months as the predictor of interest and number of retained trials as a covariate.

#### Metagenome processing

Raw metagenomic sequence reads (2.5 × 10⁷ ± 1.4 × 10⁷ reads/sample) were processed using tools from the bioBakery as previously described (17, 45). Briefly, KneadData v0.10.0 was used with default parameters to trim low-quality reads and remove human sequences (using reference database hg37). Next, MetaPhlAn v3.1.0 (using database mpa_v31_CHOCOPhlAn_201901) was used with default parameters to map microbial marker genes to generate taxonomic profiles. Taxonomic profiles and raw reads were passed to HUMAnN v3.7 to generate stratified functional profiles.

#### Microbial community analysis

Principal coordinates analysis was performed in the Julia programming language (46) using the Microbiome.jl package (47). Bray-Curtis dissimilarity (Distances.jl) was calculated across all pairs of samples, filtering for species-level classification. Classical multidimensional scaling was performed on the dissimilarity matrix (MultivariateStats.jl), and axes with negative eigenvalues were discarded.

Overall community variance explained by each axis is reported as the axis eigenvalue divided by the sum of positive eigenvalues. Pearson correlation (R) of the eigenvector with age at stool collection is also reported.

Individual taxonomic features were assessed for associations with VEP features using MaAsLin v3 (48) with default parameters. For concurrent visits, the model tested was species ∼ vep + age_months + n_trials

Where species is the relative abundance of each taxon, vep is the numerical value of the VEP feature (eg N1 latency), age_months is the child’s age at the time of stool collection, and n_trials is the number of EEG trials retained at that visit. For models comparing separate visits (Fig. 4), values for species and age_months are from the earlier visit (from which the stool sample was collected), and vep and n_trials are from the later visit (when VEP was measured). In addition, longitudinal models also included the term + age_diff, which is the number of months between stool collection and VEP measurement.

### Feature set enrichment analysis (FSEA)

Potentially neuroactive genesets were extracted from Supplementary Data set 1 from (5). Gut-brain modules provide KEGG Ortholog IDs (KOs) (49, 50), which were mapped to UniRef90 IDs using the utility mapping file provided with HUMAnN v3.1 (45). For each stool/VEP pair, logistic regression (LR) was performed, linking the presence or absence of that UniRef in a sample with each VEP feature (i.e., N1, P1, and N2 latencies and amplitudes), controlling for the age at which the stool sample was collected, the number of retained VEP trials, and the difference in age between the stool collection and VEP measurement. For concurrently collected stool and VEP comparisons (Fig. 2; Fig. S1), participants whose stool collection and VEP measurements were more than 2 months apart were excluded.

UniRef ∼ vep + age_months + n_trials,

where UniRef is the presence (1) or absence (2) of each annotated gene, vep is the numerical value of the VEP feature (eg N1 latency), age_months is the child’s age at the time of stool collection, and n_trials is the number of EEG trials retained at that visit. For models comparing separate visits (Fig. 4), values for UniRef and age_months are from the earlier visit (from which the stool sample was collected), and vep and n_trials are from the later visit (when VEP was measured). In addition, longitudinal models also included the term + age_diff, which is the number of months between stool collection and VEP measurement.

FSEA was performed on each gene set that had at least five members that were present in at least one sample in the relevant visit (17 at visit 1 and 19 at visits 2 and 3), against each of the 6 VEP features (N1, P1, and N2 latencies and amplitudes) according to the procedure set out in Subramanian et al. (51). Briefly, enrichment scores (ES) are calculated based on the rank order of z-statistics from the LR for each UniRef. A permutation test was then performed where the ES for 5,000 random samples of ranks of the same length as the gene set is calculated, and the pseudo-p value is the fraction of permutations where the permutation ES has a greater absolute value than the true ES.

Benjamini-Hochberg FDR correction was performed separately on all concurrently tested geneset/VEP feature combinations and all longitudinal geneset/VEP feature combinations. Corrected *P*-values (q-values) less than 0.2 were considered statistically significant. This high threshold for significance was chosen to maximize sensitivity (avoiding false negatives), although it permits low specificity (higher false positives), since this is a preliminary and exploratory study (52, 53). Only significant hits are included in relevant tables (Table S4, Tables 2–4)

**TABLE 2:** Longitudinal FSEA, visit 1 stool ⇨ visit 2 VEP

Gene set	Feature	Component	Enrichment	Q value
GABA synthesis	Amplitude	P1	0.3987	0.0807
		N2	0.3661	0.1129
Glutamate synthesis	Latency	N1	0.2829	0.0445
		P1	0.2625	0.0807
	Amplitude	P1	0.2316	0.1023
Glutamate degradation	Latency	P1	0.3369	0.1598
		N2	0.3252	0.1800
	Amplitude	P1	0.3256	0.1760
Tryptophan synthesis	Latency	N1	0.2211	0.0272
		P1	0.1671	0.0807
		N2	0.1900	0.0484
	Amplitude	N2	0.1630	0.1023
Quinolinic acid synthesis	Amplitude	N1	0.2735	0.1609
Quinolinic acid degradation	Latency	P1	0.1979	0.1023
		N2	0.1950	0.1118
Acetate synthesis	Latency	N1	0.2190	0.0293
		P1	0.1926	0.0611
		N2	0.2157	0.0318
	Amplitude	P1	0.1860	0.0742
		N2	0.1960	0.0549
Butyrate synthesis	Amplitude	N1	0.3193	0.1698
Isovaleric acid synthesis	Latency	N1	0.2711	0.1223
	Amplitude	N1	0.2412	0.1800
		P1	0.2409	0.1609
Menaquinone synthesis	Latency	N1	0.1393	0.1598
Inositol synthesis	Amplitude	N1	0.4295	0.1804
	Latency	N1	0.4219	0.1834
p-Cresol synthesis	Amplitude	N1	0.3665	0.1825
		P1	0.4520	0.1014
		N2	0.5164	0.0445
17-beta-Estradiol degradation	Latency	N2	0.3550	0.1800

**TABLE 3:** Longitudinal FSEA, visit 1 stool ⇨ visit 3 VEP

Gene set	Feature	Component	Enrichment	Q value
GABA synthesis	Latency	P1	0.4343	0.0445
	Amplitude	P1	0.4099	0.0742
		N2	0.5065	0.0254
Glutamate synthesis	Latency	N1	0.3035	0.0293
	Amplitude	N1	0.2344	0.1118
Tryptophan synthesis	Latency	N1	0.1875	0.0445
	Amplitude	P1	0.2176	0.0159
Quinolinic acid degradation	Latency	P1	0.1862	0.1093
		N2	0.2624	0.1834
	Amplitude	P1	0.1689	0.1609
Acetate synthesis	Latency	N1	0.1728	0.0807
Propionate synthesis	Amplitude	P1	0.3586	0.0807
Propionate degradation	Latency	N1	0.5097	0.1093
		P1	0.5345	0.0880
	Amplitude	P1	0.7388	0.0000
		N2	0.6669	0.0272
Butyrate synthesis	Latency	N1	0.3373	0.1223
		N2	0.3232	0.1498
	Amplitude	P1	0.3688	0.1014
Menaquinone synthesis	Latency	P1	0.1582	0.0870
	Amplitude	N1	0.1417	0.1397
		P1	0.1488	0.1131
		N2	0.1567	0.1023
Inositol synthesis	Amplitude	N2	0.4538	0.1210
ClpB	Latency	N1	0.2835	0.1804
	Amplitude	P1	0.3284	0.1129
		N2	0.3585	0.0800

**TABLE 4:** Longitudinal FSEA, visit 2 stool ⇨ visit 3 VEP

Gene set	Feature	Component	Enrichment	Q value
GABA synthesis	Latency	N1	0.3762	0.1073
Glutamate synthesis	Latency	N1	0.1676	0.1609
		P1	0.2979	0.0159
	Amplitude	P1	0.2342	0.0381
Glutamate degradation	Latency	P1	0.3174	0.1404
	Amplitude	N2	0.2829	0.1875
Tryptophan synthesis	Amplitude	N1	0.1554	0.0608
		P1	0.1865	0.0293
Quinolinic acid synthesis	Latency	N1	0.3186	0.0610
		P1	0.3331	0.0437
	Amplitude	P1	0.2577	0.1391
Acetate synthesis	Latency	N1	0.1673	0.0610
		P1	0.2574	0.000
		N2	0.1373	0.1529
	Amplitude	P1	0.1469	0.1210
Propionate synthesis	Amplitude	P1	0.2994	0.1262
Propionate degradation	Latency	N1	0.5503	0.0742
		N2	0.4492	0.1609
	Amplitude	P1	0.5088	0.1118
		N2	0.4229	0.1875
Butyrate synthesis	Latency	N1	0.3316	0.0807
		P1	0.3335	0.0742
		N2	0.2908	0.1223
	Amplitude	N2	0.3066	0.1118
Isovaleric acid synthesis	Latency	P1	0.2316	0.1556
	Amplitude	P1	0.2469	0.1129
Menaquinone synthesis	Latency	N1	0.1528	0.1210
	Amplitude	N1	0.1644	0.1073
		P1	0.2014	0.0293
		N2	0.1634	0.1121
Inositol degradation	Latency	N1	0.6381	0.0293
		P1	0.5323	0.1014
	Amplitude	P1	0.6413	0.0293
p-Cresol synthesis	Latency	P1	0.3118	0.1397
		N2	0.3152	0.1391
	Amplitude	N1	0.3039	0.1658
S-Adenosylmethionine synthesis	Amplitude	N1	0.2228	0.1556
		P1	0.2295	0.1347
17-beta-Estradiol degradation	Amplitude	P1	0.2818	0.1875
ClpB	Latency	P1	0.2687	0.1804

For longitudinal comparisons, all participants who had a stool sample collected at one visit and a VEP assessment at a subsequent visit were included (visit 1 stool → visit 2 VEP, *N* = 84; v1 → v2, *N* = 76; v2 → v3, *N* = 69). A total of 95 geneset/VEP features were significant when using an FDR-corrected *P*-value cutoff of q < 0.2. To ensure the robustness of these findings, we randomly permuted participant IDs between the stool and VEP assessments and repeated the analysis. Over 10 random permutations, a mean of 19.5 significant associations were identified, suggesting that FDR correction is correctly calibrating the false-positive rate.

## RESULTS

### The brain and microbiome develop rapidly in the first months of life

To investigate the co-development of the gut microbiome and visual neurodevelopment, we collected stool and the VEP in a longitudinal cohort of 194 children in South Africa during the first 18 months of life (Fig. 1A and B, Table 1; visit 1, *N* = 119, age 3.7 ± 0.9 months, visit 2, *N* = 144, age 8.6 ± 1.5 months, and visit 3, *N* = 130, age 14.1 ± 1.0 months). As expected for children at this age, both amplitude and latency VEP features were strongly correlated with age (18, 54). That is, as infants got older, N1 amplitude became more negative (N1: b = −0.07, *P* < 0.05), corrected P1 amplitude became smaller (P1: b = −0.50, *P* < 0.05), corrected N2 amplitude became smaller (N2: b = 0.52, *P* < 0.05), and all latencies became shorter (N1: b = −0.79, *P* < 0.05; P1: b = −1.21, *P* < 0.05; N2: b = −4.10, *P* < 0.05) (Fig. 2A and B).

Similarly, microbial composition was developmentally dependent, as expected (54–56). Ordinations reveal a similar relationship with age, with the first principal coordinate axis for both taxonomic profiles (Fig. 2C; variance explained = 15.1%; R = −0.50) and functional profiles (Fig. 2D; variance explained = 12.9%; R = −0.57) driven strongly by the age of the participant at the time of collection. Early samples were dominated by *Bifidobacterium* and *Bacteroides* species, whereas later samples have increasing *Prevotella* and anaerobic genera such as *Faecalibacterium* (Fig. 2E). However, individual taxa were generally not associated with VEP features after controlling for age; we analyzed taxonomic profiles for associations with each VEP feature at each visit using MaAsLin (48), and the only species to pass FDR correction was *F. prausnitzii*, which was negatively associated with P1 amplitude at visit 3 (Table S5).

### Microbial genes with neuroactive potential are associated with concurrently measured visual development

To test whether microbial metabolic potential was related to early life brain activity, we performed feature set enrichment analysis (FSEA) using previously defined groups of potentially neuroactive microbial genes and the concurrently measured VEP amplitude and latency features (5, 17). For each gene set that had at least five genes represented in a given comparison group, logistic regression was performed using VEP features (corrected for previous visit values in the case of visits 2 and 3, see methods) as predictors and the presence or absence of each microbial gene in the metagenome as the response to determine concurrent associations (see Methods). Z statistics for in-set genes were compared with all genes using a permutation test to determine the significance of the associations (51).

**Fig 3:**
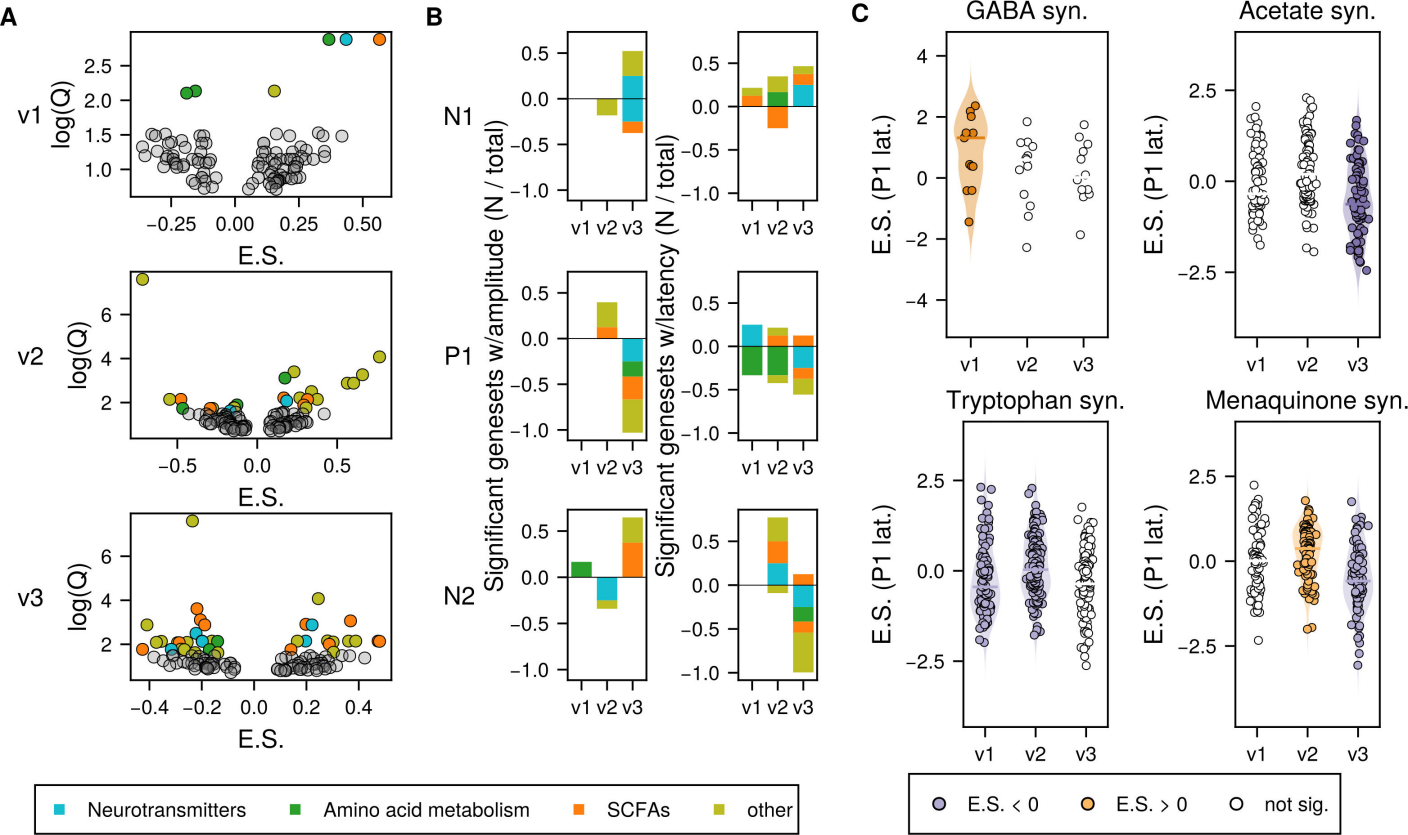
Feature set enrichment analysis reveals associations between microbial genes and VEP. (**A**) Volcano plots of gene sets tested with feature set enrichment analysis (FSEA) for all 6 VEP features for each visit (visit 1 top, visit 2 middle, and visit 3 bottoms) with enrichment score (E.S.) compared with log scaled FDR-corrected *P*-value (Q). Colored dots were significantly enriched (positive E.S.) or depleted (negative E.S.) relative to the tested VEP feature. All VEP feature/gene set combinations are represented here, see Fig. S1 for disambiguated results. (**B**) Summary of results in (**A**), showing the fraction of each class of neuroactive genes (neurotransmitter metabolism, SCFA metabolism, amino acid metabolism, or other) that were statistically significantly enriched (bars above 0) or depleted (bars below 0) for each VEP feature for each visit in the analysis. (**C**) Enrichment plots for selected gene sets and their association with concurrently measured P1 latency. Each plot shows the distribution of associations of individual genes within the gene set and the VEP feature. Dots are colored if the geneset as a whole is significantly associated. Enrichment plots for all gene set / VEP feature associations are shown in Fig. S1.

Of the 35 genesets assessed, 17 had sufficient representation to test at visit 1, and 19 were tested at visits 2 and 3. Of these, 18 were significantly associated with at least one VEP feature during at least one visit within the 18-month window, after correcting for false discovery rate (Benjamini-Hochberg, q < 0.2; Fig. 3A and B, Tables S4 and 6, only significant hits are included). Microbial genes involved in the synthesis or degradation of molecules with neuroactive potential across all categories considered (i.e., neurotransmitters, amino acid metabolism, SCFAs, and others) were associated with both concurrent VEP amplitudes and latencies at each visit (Fig. S1), demonstrating widespread associations between early life gut microbiome and visual cortex neurodevelopment. The number of these concurrent associations increased over time (visit 1 had six associations, visit 2 had 24, and visit 3 had 37).

Specifically, across the gene sets involved in neurotransmitter synthesis and degradation, glutamate synthesis/degradation and GABA synthesis showed associations with all VEP features, primarily at the second and third visits (mean ages 8.6 and 14.1 months, respectively; Fig. 3C; Table S4). Gene sets involved in tryptophan metabolism and associated pathways (i.e., quinolinic acid) were also strongly concurrently related to VEP development.

Several short-chain fatty acid (SCFA)-metabolizing gene sets were also found to have multiple associations with VEP features. Specifically, acetate synthesis was strongly associated with almost all VEP features (Table S4). Butyrate synthesis was associated with P1 and N2 amplitudes and latencies from 6 months onward (visits 2 and 3), when the visual cortex is most actively undergoing myelination. Finally, propionate synthesis/degradation was significantly associated with VEP latencies at every visit over the 18-month window (N1 at visits 1 and 2, and both P1 and N2 at visit 3). These SCFA-metabolizing genes showed almost double the concurrent associations with VEP latencies than amplitudes (11 associations with latencies and six associations with amplitudes).

Finally, within the remaining gene sets tested, we observed robust associations in particular between menaquinone (Vitamin K2) gene sets and the VEP features over this infancy window. This is an expected relationship, as vitamin K2 specifically is posited to promote healthy vision, both outside of the brain through effects on the retina, and within the brain where it can protect neural circuits from oxidative stress (57).

Notably, across significant gene set associations with VEP features, the P1 and N2 component amplitudes and latencies were consistently the most sensitive to these microbial gene sets. Both P1 and N2 components are known to show the most protracted and dramatic changes with development during the first year of life (58) and may best reflect underlying visual learning and plasticity at this stage (Table S7).

### Microbial metabolic potential predicts future brain development in infancy

**Fig 4:**
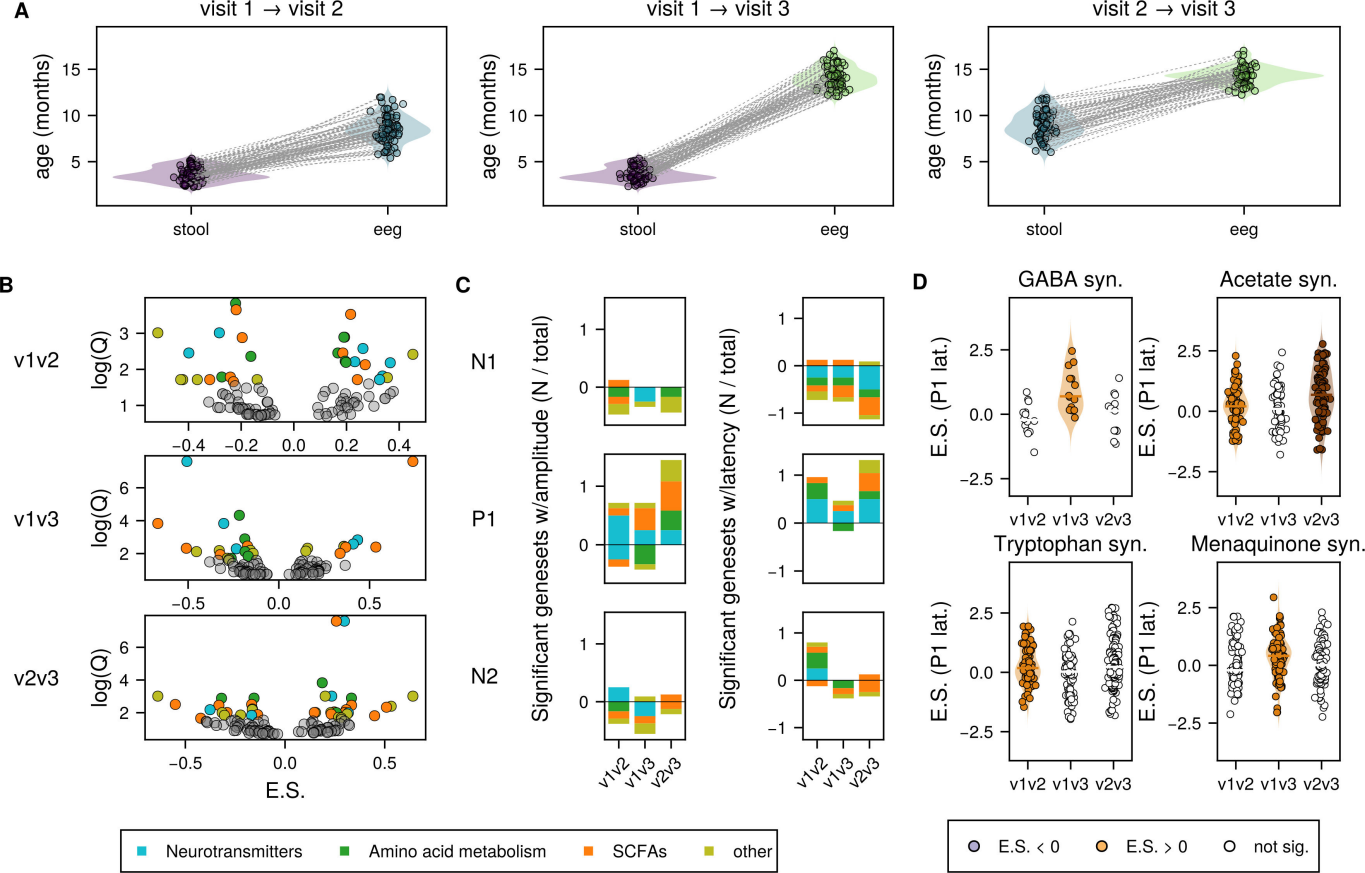
Early gut microbial metabolism is associated with future VEP development. (**A**) age distributions for cross-visit comparisons, with age at stool collection (left) and age of VEP measurement (right) for each participant included in the analysis. Collections for the same individual are connected by a dotted gray line. (**B**) Volcano plots of gene sets tested with feature set enrichment analysis (FSEA) for all 6 VEP features, with enrichment score (E.S.) compared with log scaled FDR-corrected *P*-value (Q). Colored dots were significantly enriched (positive E.S.) or depleted (negative E.S.) relative to the tested VEP feature. (**C**) Summary of results in (**B**), showing the fraction of each class of neuroactive genes (neurotransmitter metabolism, SCFA metabolism, amino acid metabolism, or other) that were statistically significantly enriched (bars above 0) or depleted (bars below 0) for each VEP feature for each cross-visit comparison in the analysis. (**D**) Enrichment plots for selected gene set and their association with P1 latency. Each plot shows the distribution of associations of individual genes within the gene set and the VEP feature. Dots are colored if the geneset as a whole is significantly associated. Enrichment plots for all gene set/VEP feature associations may be found in Fig. S1.

We initially hypothesized that the earliest microbial influences would have the largest effects on brain development, but in cross-sectional analysis with concurrently measured VEP, we observed the fewest number of associations at visit 1. To differentiate whether this cross-sectional finding indicated the early microbiome was sparsely related to visual cortical development or instead took time to manifest its influence, we sought to determine whether microbial genes at early time points were associated with later VEP development. We therefore performed FSEA on stool samples collected at visit 1 with visit 2 VEP (Table 2, age at stool collection = 3.6 ± 0.8 months, age at VEP = 8.6 ± 1.5 months) or visit 3 VEP (Table 3, age at stool collection = 3.7 ± 0.7 months, age at VEP = 14.1 ± 1.1 months), as well as visit 2 stool samples with visit 3 VEP (Table 4, age at stool collection = 8.9 ± 1.5 months, age at VEP = 14.3 ± 1.0 months; Fig. 4, Table 4, Table S8).

All gene sets that had a significant hit with concurrently measured VEP were also significantly associated with at least one future VEP feature, except those involved in the synthesis of 3,4-dihydroxyphenylacetic acid (DOPAC), a metabolite of dopamine (Fig. S2B, Tables 2–4). Notably, the quantity of those associations increased substantially for all longitudinal comparisons compared with concurrent comparisons. For example, only six visit 1 microbial gene sets were associated with visit 1 VEP features, and each of those was only associated with a single concurrently measured VEP feature. By contrast, these longitudinal analyses revealed that visit 1 microbial gene sets showed a much richer pattern of associations with future VEP feature development. Specifically, 13 of the 17 gene sets that were tested were associated with visit 2 VEP features, and 11 were associated with visit 3 VEP features. Of these, the majority (9/13 for visit 2, 8/11 for visit 3) were associated with at least 2 VEP features, and nearly half (6/13 for visit 2, 5/11 for visit 3) were associated with more than two future VEP features (Fig. 4B and C).

Longitudinally, the early microbiome (visit-1) was related to VEP features at visit 2 and visit 3 fairly evenly; of 12/28 significant gene set/VEP latency tests, 16 were from visit 2 and 12 were from visit 3, and of the 30 significant associations with VEP amplitudes, 15 were from visit 2 and 15 were from visit 3. This suggests that early microbiome metabolism in the first 6 months of life is associated with visual neurodevelopment over the next year. Microbiome metabolism from visit 2 was associated with similar numbers of visit 3 VEP features as visit 1 microbiome (17 visit 3 latency features, 18 visit 3 amplitude features), suggesting continued co-development of these systems over the first postnatal year. Neurotransmitters GABA and glutamate, tryptophan metabolism (tryptophan and quinolinic acid), SCFAs including acetate, butyrate, and propionate, as well as menaquinone (Vitamin K2) were again all significantly associated with multiple VEP features across multiple longitudinal comparisons.

Importantly, the nature and identity of the longitudinal associations varied over development for many gene sets, indicating temporal specificity to these associations. With respect to the neurotransmitter-related pathways, among the associations between GABA synthesis genes and future VEP features, GABA genes specifically from visit 1 showed the greatest number of associations with future VEP features (5/6 GABA associations) at visits 2 and 3 equally (Fig. 4D, Fig. S2), and the majority of these associations were VEP amplitudes (reflecting development of neurotransmission including excitatory/inhibitory balance). To a lesser extent, glutamate metabolism genes followed a similar temporal pattern (8/13 glutamate associations involved visit 1 gene) but did not relate to amplitudes or latencies differentially like GABA. This pattern of results suggests early (within the first six postnatal months) microbiome GABA/glutamate dynamics, especially GABA, are most relevant for changes to visual cortex function over the following year.

Tryptophan-related pathway genes (responsible for generating serotonin, among other products) from visit 1 were also responsible for the majority of associations with future VEP features (tryptophan: 6/8 associations; quinolinic acid: 6/9 associations, Fig. 4D, Fig. S2). In contrast to GABA but similar to glutamate, tryptophan-related gene associations were largely shorter-term associations with VEP features at the visit immediately following gene set measurement (approximately 5 months later; 12/17 associations), indicating dynamic co-development over the first 18 months of life. Across neurotransmitter-related gene set associations (GABA, glutamate, tryptophan/serotonin), there was thus a clear pattern whereby early (approximately 4 months old) microbiome gene sets showed the largest number of associations with subsequent VEP feature development.

SCFAs showed a different developmental pattern of associations with future VEP features. Specifically, propionate and butyrate metabolism genes from both visit 1 and visit 2 showed associations with future VEP features, but here, the effects were almost entirely observed for VEP features at visit 3 (10/10 propionate and 7/8 butyrate associations, Fig. 4D, Fig. S2). Moreover, acetate and butyrate metabolism genes were doubly associated with future VEP latencies compared with amplitude features.

Finally, menaquinone (Vitamin K2) metabolism genes followed a similar pattern to the SCFAs in that genes from visit 1 and visit 2 were largely associated with future VEP features at visit 3 (8/9 menaquinone associations). This indicates persistent associations of this early microbiome gene set with individual differences in VEP features early in the second year of life.

It is possible that extrinsic factors related to development mutually influence both the gut microbiome and neural development, although we additionally tested whether VEP features were associated with microbial metabolism at a future visit and found substantially fewer associations (29 total associations, compared with 95 when analyzing early stool samples with future VEP). Although this does not prove a causal relationship, it is consistent with the hypothesis that microbial metabolism influences brain development.

## DISCUSSION

The past decade has seen remarkable growth in our understanding of the relations between the gut microbiome and the brain. However, a great deal of that investigation has focused on adult populations or neuropsychiatric disorders, limiting the potential to explain how and when these associations emerge during development. Here, we address this key open question by leveraging a rich longitudinal data set over the first year and a half of life, which is the time of greatest developmental change for both the microbiome and brain, given the unfolding of foundational sensory neurodevelopment. Our data revealed that microbial genes involved in the metabolism of neuroactive molecules are associated with concurrent and subsequent visual cortical neurodevelopment. These pathways included those for the neurotransmitters GABA and glutamate, the amino acid tryptophan, and short-chain fatty acids involved in myelination, including acetate and butyrate.

Specifically, we have shown a robust, prospective relationship between microbial genes involved in the metabolism of neuroactive compounds and the development of visual cortical function as measured by the VEP electrophysiological response. We found that microbial metabolism is more strongly associated with future measures of the VEP than those collected concurrently. Although not dispositive, this would be the predicted outcome if microbial genes are causally influencing brain development. As additional evidence for this interpretation, we did not observe the same rich set of associations in the converse analyses examining whether VEP related to future microbiome properties. Microbial metabolism within the first 6 months shows the most associations with subsequent visual neurodevelopment, suggesting the early postnatal microbiome may play a particularly important role in the co-development of these systems. This interpretation is also supported by prior research showing that associations of the microbiome with behavioral readouts of neurocognition are stronger prospectively than concurrently (59). Moreover, specific associations between gene sets and VEP features showed temporal specificity within the 18-month developmental window assessed, suggesting that the impact of early microbial metabolism on the brain is developmentally dependent.

Notably, the gene sets most highly associated with visual functional neurodevelopment over infancy are for the metabolism of molecules with known links to developmental neuroplasticity (60–62). Specifically, we observed associations for gene sets related to glutamate and GABA, neurotransmitters that are central to regulating excitatory/inhibitory (E/I) cortical balance.

Developmental changes in E/I balance modulate the degree of neuroplasticity in the mammalian cortex, including regulating the start and progression of critical period neuroplasticity mechanisms in the visual cortex (28, 29, 60, 61). Our observed pattern of results suggests early (within the first 6 postnatal months) microbiome GABA/glutamate dynamics, especially GABA, are most relevant for changes to visual cortex function over the following year. Gut production of GABA may influence cortical GABA levels via active transport from the bloodstream to the brain (63–65). Recent evidence suggests that gut-derived glutamate may also influence brain levels and function (8–10) and can operate via indirect mechanisms (either transformation into GABA or via regulating glutamate levels in the bloodstream that impact glutamate transfer from the brain to the bloodstream).

Tryptophan-related pathway genes were also identified here that are responsible for generating serotonin as well as other neuroactive molecules such as kynurenic acid (an SMDAR antagonist) (12). Both serotonin and kynurenic acid are implicated in early neuroplasticity and neurotransmitter regulation, and serotonin has potent effects on visual cortex plasticity in particular (66, 67). Although quinolinic acid is part of the kynurenine pathway and is a neurotoxin that can cause neuronal dysfunction, it may also play a role in glutamate uptake in the brain (68, 69). Specifically, tryptophan metabolism genes were associated with VEP latencies just after each VEP component showed its greatest window of developmental change (components emerge sequentially as follows: P1, N1, and N2). This pathway may thus relate to processes stabilizing the neural circuitry (i.e., downregulating neuroplasticity) underlying each VEP component, an account consistent with recently observed effects of serotonin within the visual cortex in rodents (70). Importantly, nearly all of the body’s serotonin is produced in the gut by enterochromaffin cells, and this biosynthesis is regulated by microbes (71, 72), making this pathway an especially promising candidate intervention target for future research in development.

We further found that gene sets for short-chain fatty acids important in downregulating neuroinflammation and promoting myelination within the brain were robustly related to visual neurodevelopment. Myelination is important for downregulating plasticity in neural circuitry overdevelopment by stabilizing and protecting circuits that have been shaped by early experience (73). Specifically, we observed associations between acetate, butyrate, and propionate genes with VEP development. Acetate is a critical component required for the increased lipid synthesis that happens during postnatal myelination in the brain (74). Circulating butyrate also increases myelination (75), and although propionate’s relation to myelinating oligodendrocytes remains unclear, it is known to protect myelinating Schwann cells outside of the brain from oxidative stress (76). Acetate, butyrate, and propionate are all also widely regarded as neuroprotective by promoting healthy microglial development and downregulating neuroinflammation that interferes with myelination (77). VEP latency features reflect myelination (29, 78, 79), and accordingly, these SCFAs showed more associations with VEP latency features prospectively, especially VEP latency features in visit 3. This pattern of results is consistent with these SCFA roles in myelination occurring over the second half of the developmental window studied. SCFAs including acetate, butyrate, and propionate can pass the blood-brain barrier to directly influence myelination-related processes within the brain. Taken together, the pattern of results across GABA, glutamate, tryptophan, and SCFA gene sets suggests that early postnatal microbiome-derived metabolites relate to key neuroplasticity regulation processes within the cortex.

Recent global-scale studies (80) have shown that gut microbiome maturation follows a normative, stage-like trajectory, with compositional and functional shifts occurring in age-linked patterns across diverse populations. These shifts result in different microbial communities and metabolic capacities being dominant at different stages of infancy. In this context, our longitudinal findings, in which different microbial gene sets are associated with neurodevelopmental features at different time points, are especially notable. The observation here that early associations (e.g., GABA metabolism) differ from later ones (e.g., SCFA production) aligns with known transitions from *Bifidobacterium*-dominated, human milk oligosaccharide-focused metabolism in early infancy to later functions like amino acid fermentation and SCFA synthesis as taxa such as *Faecalibacterium prausnitzii* rise in abundance. These longitudinal patterns suggest that different microbiome-derived metabolites may exert their strongest neurodevelopmental influence at distinct stages—each coinciding with predictable phases of microbial functional maturation.

Brain structure and function are sensitive and responsive to early-life environmental conditions. Variations in myelination and neural connectivities have been associated with malnutrition, chronic stress, exposure to violence, access to sanitation, and alterations in the gut microbiome (81, 82). It is thus critical to study child development in diverse geographical and cultural contexts. Our infant cohort was recruited from Gugulethu, an urban settlement near Cape Town, South Africa. Gugulethu is characterized by a mix of formal and informal housing, many families live in economically disadvantaged conditions, with limited access to sanitation and other basic services. Although breastfeeding is widely promoted in this community, some infants are introduced to complementary foods such as porridge and cereals before the recommended 6 months of age. These early feeding practices differ substantially from those in higher-income or industrialized settings, where prolonged breastfeeding and formula feeding are more common. In addition to diet, other factors, such as sanitation, maternal education, maternal depression, maternal mental health, and overall household environment, also differ from many settings in the global North and likely contribute to a distinct microbial and environmental exposure landscape. Therefore, studying infant development in this context enables us to identify both universal and context-specific features of gut microbial development and their potential influence on neurodevelopment.

Our study is a substantial advancement over prior work on the microbial-gut-brain axis in early life due to the sequencing method, the large number of participants, the longitudinal study design, and the inclusion of participants from a scientifically under-represented region of the world. The use of shotgun metagenomic sequencing enables direct interrogation of microbial metabolic potential. Prior research primarily used amplicon (16S rRNA gene) sequencing, which enables lower-resolution taxonomic identification and is restricted to inferring metabolic potential based on taxonomy. This was particularly important in this study, since the investigation of taxonomic profiles revealed that only a single microbe (*F. prausnitzii*) was associated with any VEP feature at any visit we tested. Moreover, several studies in infancy have inferred gut-brain associations by linking microbiome measures to subsequent neurodevelopmental measures using behavioral assessments (e.g., Bayley Scales of Infant Development), noting associations with visually mediated cognition (59). However, this study assessed gut-brain associations directly using the VEP derived from electroencephalography. The VEP is advantageous because features reflect largely neurotransmission-related (via amplitudes) or structural (i.e., myelination, via latencies) changes over this developmental window, facilitating some specificity in the observed associations. Moreover, the VEP can be indexed with fidelity from birth, providing a continuous measure of visual cortical function across the study age range. Additionally, this study involved a large number of participants (194) contributing dense longitudinal data, with up to three time points, all taken in the first 18 months of an infant’s life. Although prior work focused on single time point measures of microbiome and neurodevelopment (83), longitudinal associations allowed us to investigate the changing relation between gut microbial metabolism and the development of visual neurocircuitry over time.

One limitation of this study is the fact that we are only able to observe the genomic composition of the microbiome, rather than the concentration of metabolites themselves. This prevents us from determining the concentration of these molecules in the gastrointestinal tract, blood, and brain, as the abundance of these genes does not provide information about their activity, their interactions with other metabolic pathways (including those of the host), or absorption by colonic epithelial cells.

Moreover, the relationship between gene abundance and molecule concentration may be counterintuitive, since the relationship between degradation and synthesis of metabolites occurs both at the individual organism level and at the community level. For example, genes for breaking down a molecule may be prevalent if that molecule is at high concentrations, or the molecules may be rapidly degraded by other members of the community the moment they are produced. Furthermore, it may be that the relation between metabolite and brain development remains stable over time, but the relation between molecules and microbial selection changes at different stages of life. Addressing these limitations in humans is challenging, even if looking at stool metabolites, because overall exposure throughout the gastrointestinal tract is not necessarily reflected in the final concentration of those molecules in the stool. Therefore, metabolites from blood plasma could provide more accurate systemic concentrations of molecules, but challenges remain on how to interpret them in humans (84, 85). Further computational modeling of community-scale metabolism may also yield important insights, although, to our knowledge, this has not previously been done in infant populations (86, 87).

Given that the VEP is evolutionarily conserved in mammals and can be accurately measured during development, the hypotheses generated in humans in this study are readily testable mechanistically using VEP derived from electroencephalography *in vivo* models in future research. For example, the VEP could be assessed in germ-free or defined-microbiome animals (88, 89) and may be supplemented with specific molecules such as SCFAs or colonized with microbial species lacking or providing specific metabolic pathways. Furthermore, molecule concentrations in tissues from the gut to the brain can be directly assessed in these models. Uncovering relations between microbial metabolism and specific molecules may also generate hypotheses that can be confirmed in human data. This study, therefore, provides a foundation for deep investigation of the link between the human gut microbiome and brain development.

## Data Availability

The code for initial processing of data and for analyses performed in this manuscript is available on GitHub and archived on Zenodo (90). Input data have been archived on Dryad and are downloadable via the included scripts in the analysis code. The raw sequencing data for the Khula study have been deposited in the NCBI Sequence Read Archive (SRA) under BioProject accession number PRJNA1128723. All other relevant data supporting the key findings of this study and instructions are available within the article and its Supplementary Information files.

## SUPPLEMENTAL MATERIAL

Supplemental Figure mbio.00835-25-s0001.pdf Figures S1–S8.

Table S1 mbio.00835-25-s0002.docx HAPPE preprocess parameters.

Table S2 mbio.00835-25-s0003.docx HAPPE GenerateERPs parameters.

Table S3 mbio.00835-25-s0004.docx EEG quality control.

Table S4 mbio.00835-25-s0005.xlsx Concurrent associations between neuroactive gene sets and VEP.

Table S5 mbio.00835-25-s0006.xlsx Feature-wise association of microbial species with VEP features.

Table S6 mbio.00835-25-s0007.docx Demographic information for concurrent analyses.

Table S7 mbio.00835-25-s0008.docx Age-related changes in VEP features.

Table S8 mbio.00835-25-s0009.docx Demographic information for predictive analyses.

ASM does not own the copyrights to Supplemental Material that may be linked to, or accessed through, an article. The authors have granted ASM a non-exclusive, world-wide license to publish the Supplemental Material files. Please contact the corresponding author directly for reuse.
