# Supplementary material for: Codevelopment of gut microbial metabolism and visual neural circuitry over human infancy
Source: mBio. 2025 Jun 30;16(8):e00835-25. doi: 10.1128/mbio.00835-25 (PMC12345167; doi:10.1128/mbio.00835-25)
Supplement: Supplemental Figure — Figures S1–S8. [file mbio.00835-25-s0001.pdf]

Co-development of gut microbial metabolism and visual neural circuitry over human infancy

Supplementary Figures

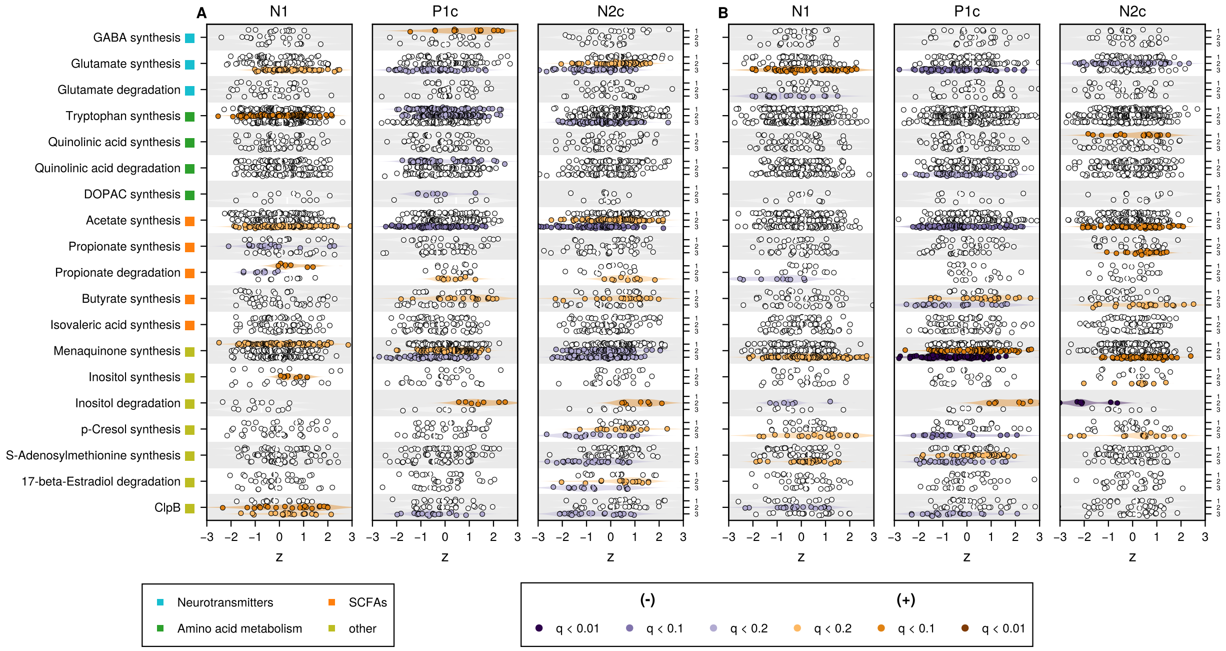

Figure S1. Concurrent feature set enrichment analysis of microbial neuroactive genes and VEP for three visits. FSEA results for all gene sets where at least one visit had a significant hit ($q < 0.2$) with at least one VEP latency (A) or amplitude (B). Dots indicate the Z-statistic from logistic regression for each gene in a gene set. Vertical bars indicate the median Z-statistic for the gene set as a whole. Y-axis position for each gene set indicates the visit number. Visit 1 for inositol degradation and DOPAC synthesis was not tested, since there were fewer than 5 genes from those gene sets present in the sample (See Methods).

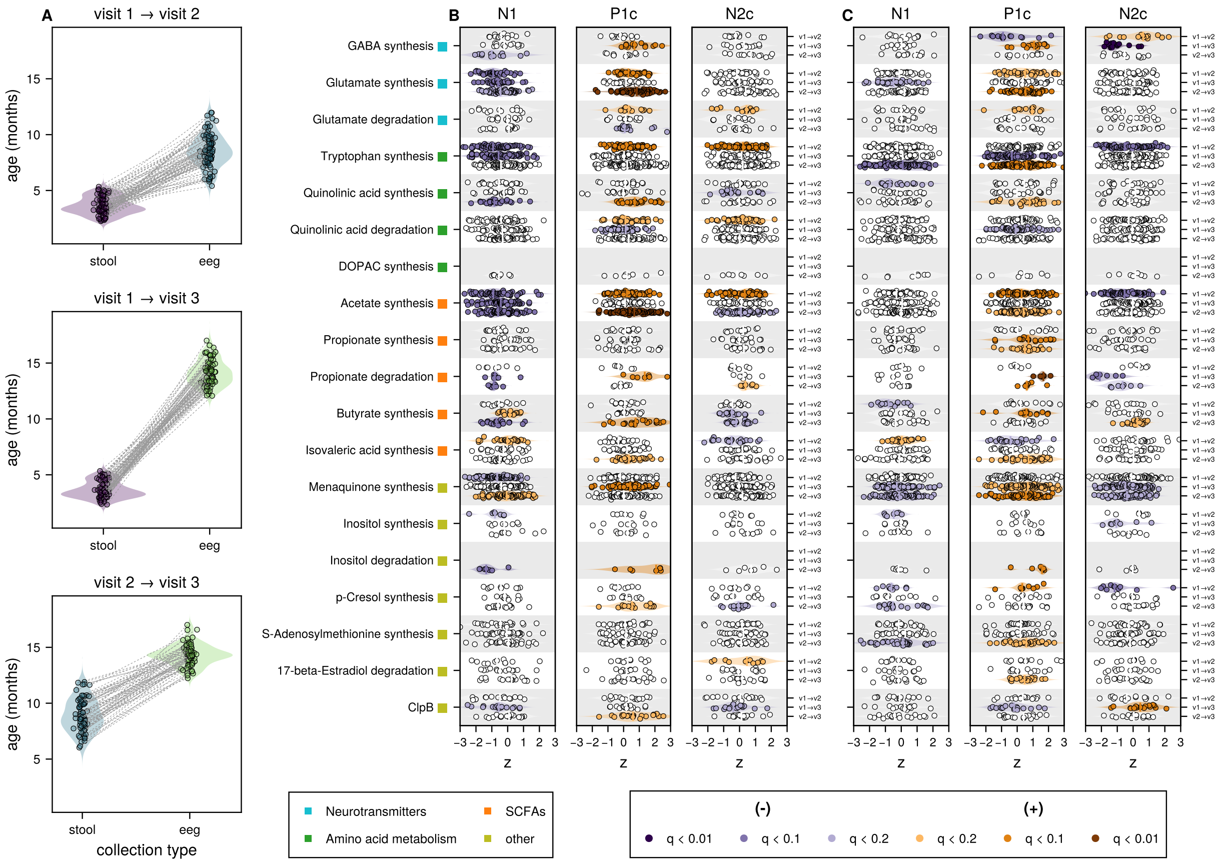

Figure S2. Gut microbial genes predict future VEP latencies and amplitudes. (A) Age distributions for stool samples (left) and VEP (right) for each longitudinal comparison (same individual) tested, V1 stool →V2 VEP, V1 stool →V3 VEP, and V2 stool →V3 VEP. V1 = visit 1, V2 = visit 2, and V3 = visit 3. As in Figure S2, (B) and (C) show FSEA results for all gene sets where at least one visit had a significant hit (q < 0.2) with at least one VEP latency or amplitude, respectively. Dots indicate the Z-statistic from logistic regression for each gene in a gene set. Vertical bars indicate the median Z-statistic for the gene set as a whole. The Y-axis position for each gene set indicates longitudinal comparison. V1 → V2 and V1 → 3 for inositol degradation and DOPAC synthesis were not tested, since there were fewer than 5 genes from those gene sets present in the sample (See Methods).

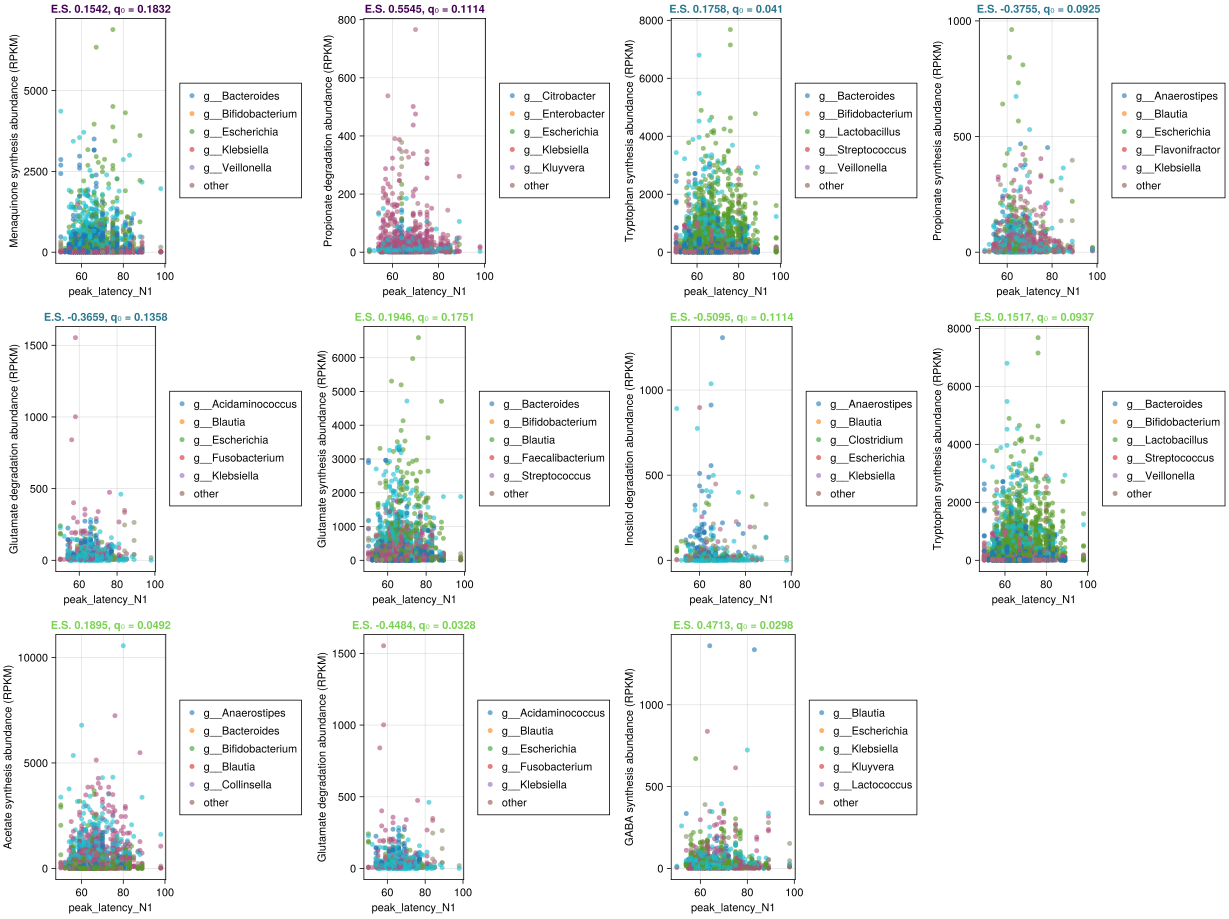

Figure S3. Taxa contributing neuroactive genes to pathways significantly associated with N1 latency. For gene sets significantly associated with N1 latency, the sum of reads per kilobase per million reads (RPKM) for all UniRef90s in that gene set was calculated for each genus. If more than 5 genera contributed UniRefs, contributions from the top 5 genera were plotted, and other genera were summed and plotted together. Titles of each plot are colored by visit, purple for Visit 1, blue for Visit 2, and green for Visit 3 (see Figure 1).

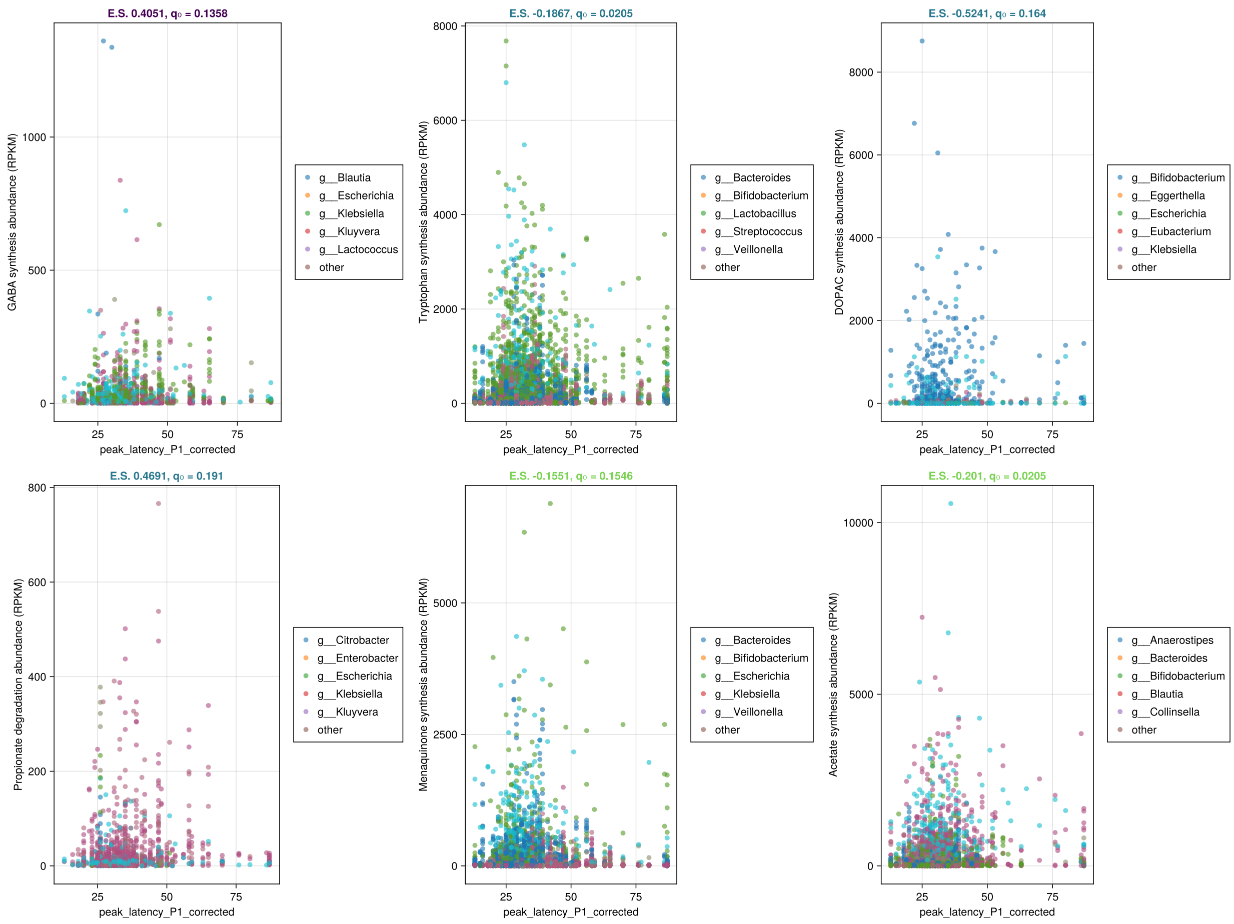

Figure S4. **Taxa contributing neuroactive genes to pathways significantly associated with P1 latency.** For gene sets significantly associated with P1 latency, the sum of reads per kilobase per million reads (RPKM) for all UniRef90s in that gene set was calculated for each genus. If more than 5 genera contributed UniRefs, contributions from the top 5 genera were plotted, and other genera were summed and plotted together. Titles of each plot are colored by visit, purple for Visit 1, blue for Visit 2, and green for Visit 3 (see Figure 1).

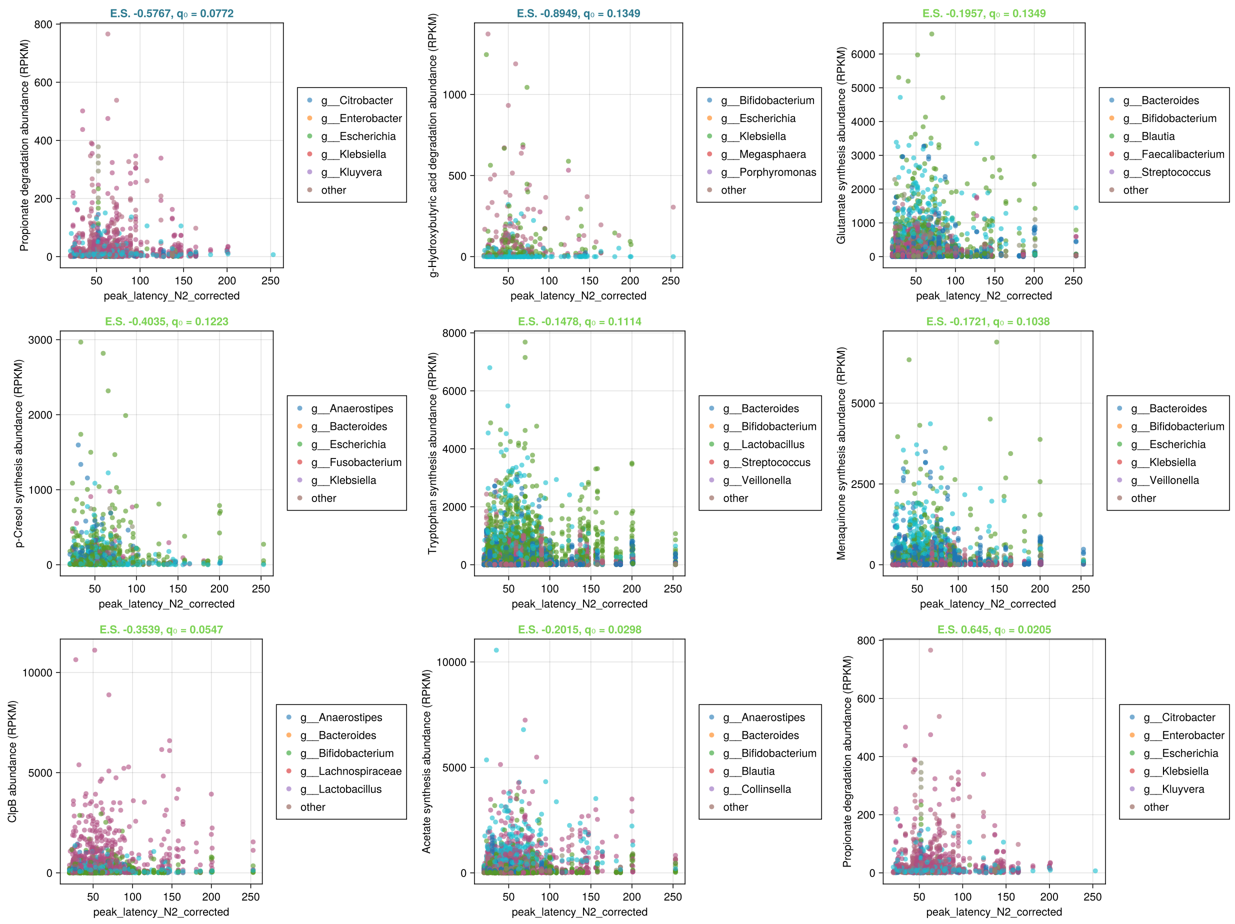

Figure S5. Taxa contributing neuroactive genes to pathways significantly associated with N2 latency. For gene sets significantly associated with N2 latency, the sum of reads per kilobase per million reads (RPKM) for all UniRef90s in that gene set was calculated for each genus. If more than 5 genera contributed UniRefs, contributions from the top 5 genera were plotted, and other genera were summed and plotted together. Titles of each plot are colored by visit, purple for Visit 1, blue for Visit 2, and green for Visit 3 (see Figure 1).

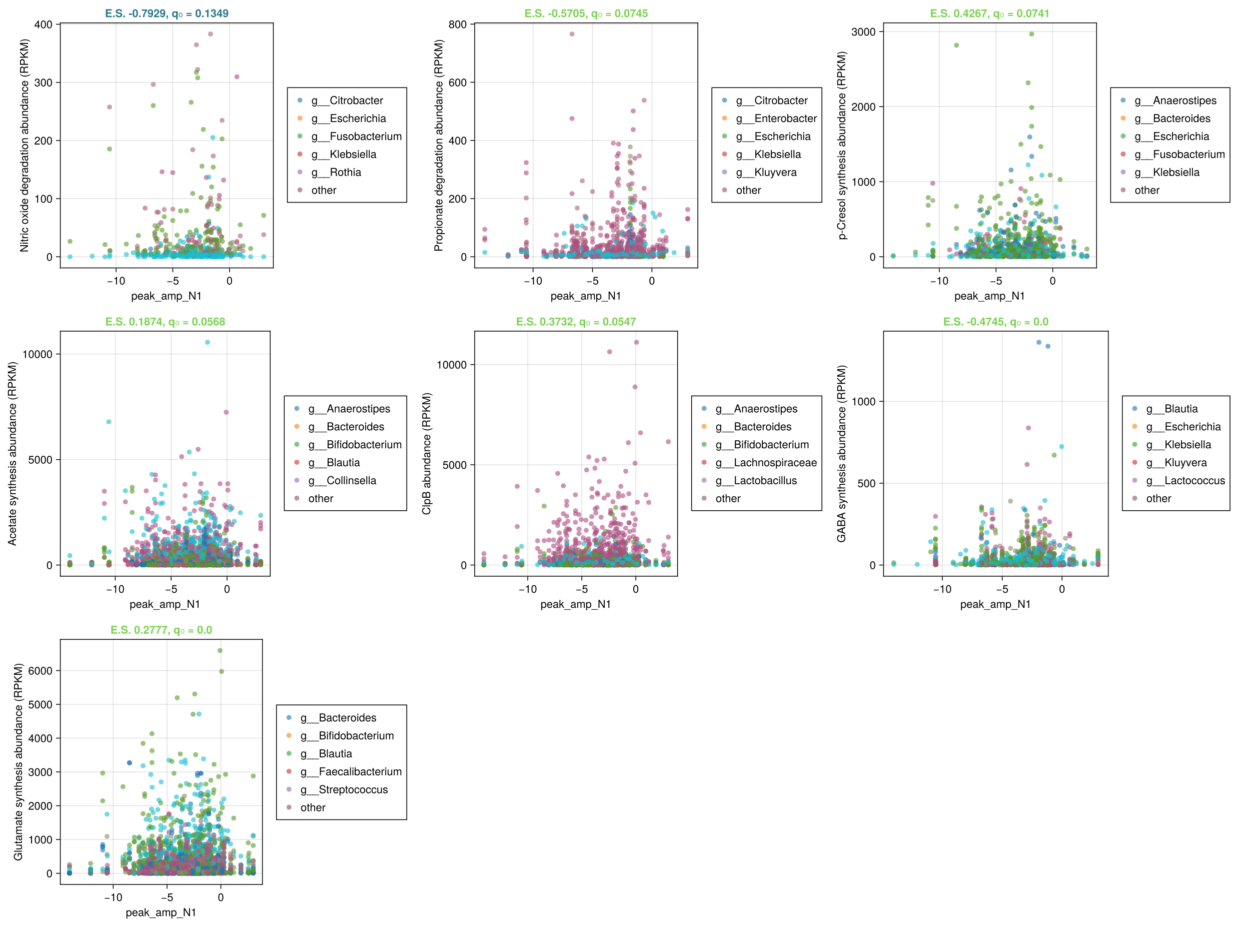

Figure S6: Taxa contributing neuroactive genes to pathways significantly associated with N1 amplitude. For gene sets significantly associated with N1 amplitude, the sum of reads per kilobase per million reads (RPKM) for all UniRef90s in that gene set was calculated for each genus. If more than 5 genera contributed UniRefs, contributions from the top 5 genera were plotted, and other genera were summed and plotted together. Titles of each plot are colored by visit, purple for Visit 1, blue for Visit 2, and green for Visit 3 (see Figure 1).

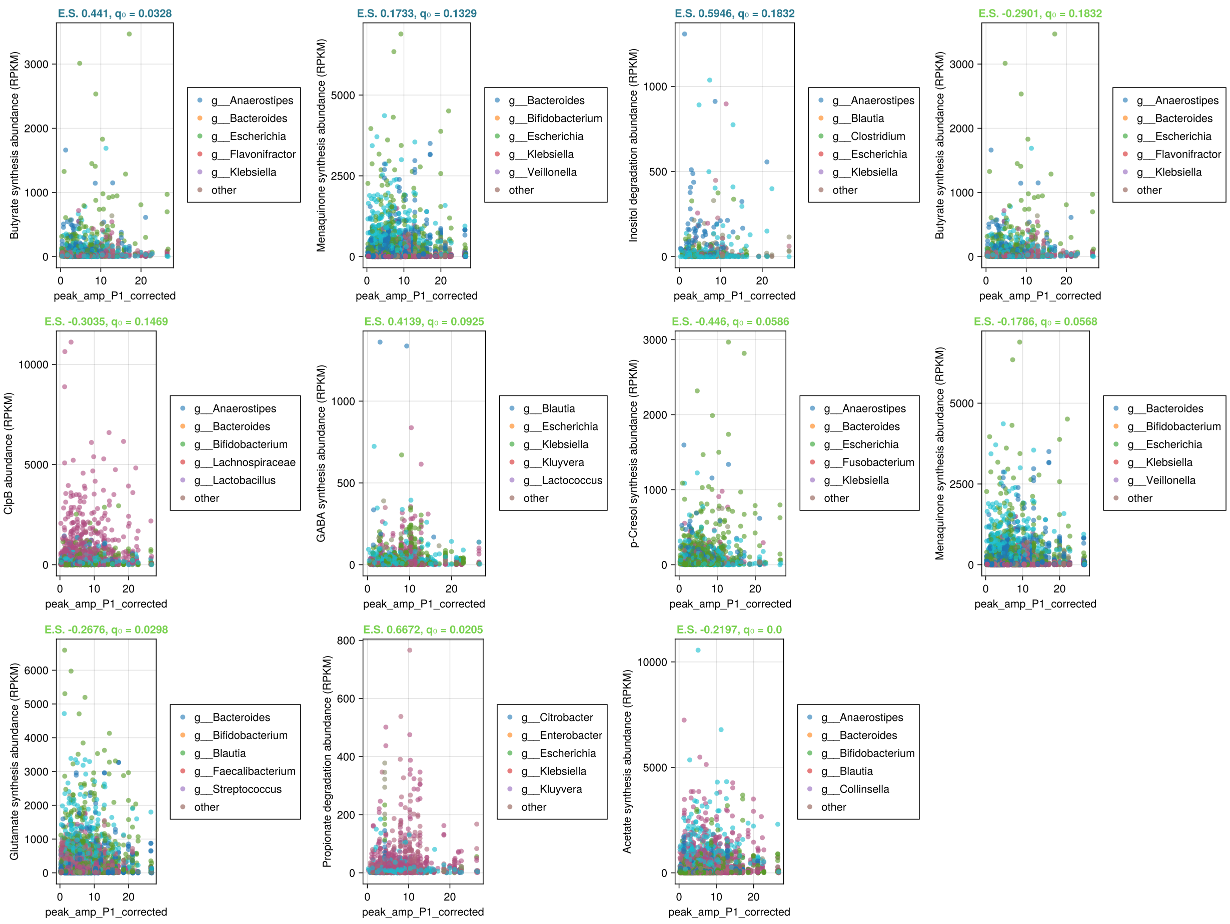

Figure S7: Taxa contributing neuroactive genes to pathways significantly associated with P1 amplitude. For gene sets significantly associated with P1 amplitude, the sum of reads per kilobase per million reads (RPKM) for all UniRef90s in that gene set was calculated for each genus. If more than 5 genera contributed UniRefs, contributions from the top 5 genera were plotted, and other genera were summed and plotted together. Titles of each plot are colored by visit, purple for Visit 1, blue for Visit 2, and green for Visit 3 (see Figure 1).

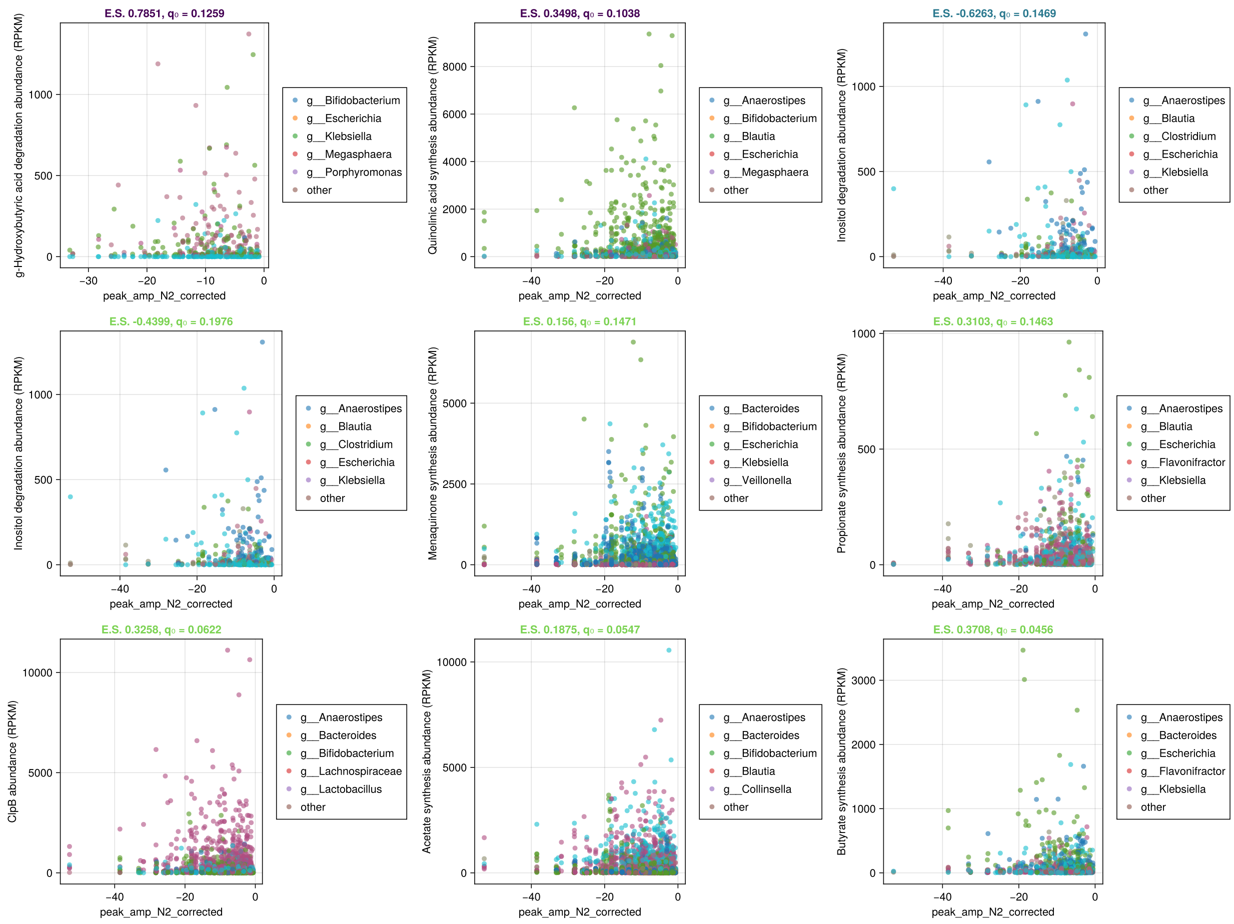

Figure S8: Taxa contributing neuroactive genes to pathways significantly associated with N2 amplitude. For gene sets significantly associated with N2 amplitude, the sum of reads per kilobase per million reads (RPKM) for all UniRef90s in that gene set was calculated for each genus. If more than 5 genera contributed UniRefs, contributions from the top 5 genera were plotted, and other genera were summed and plotted together. Titles of each plot are colored by visit, purple for Visit 1, blue for Visit 2, and green for Visit 3 (see Figure 1).
